# Mediated effect of white blood cell on the association between 1-hydroxynaphthalene and the prevalence of depression: Evidence from the NHANES (2005–2016)

**DOI:** 10.1097/MD.0000000000045477

**Published:** 2025-11-14

**Authors:** Liuyan Xu, Qingsong Mao, Xiaoyi Zhu, Xinyi Zhang, Yuzhe Kong

**Affiliations:** a Department of Infection Control, The Fourth Affiliated Hospital of School of Medicine, and International School of Medicine, International Institutes of Medicine, Zhejiang University, Yiwu, China; b Hepatobiliary Pancreatic Surgery, Banan Hospital Affiliated of Chongqing Medical University, Chongqing, China; c Xiangya School of Medicine, Central South University, Changsha, China; d College of Education, Wenzhou University, Wenzhou, China.

**Keywords:** 1-hydroxynaphthalene, depression, inflammation factors, NHANES, white blood cell

## Abstract

This study investigated the impact of exposure to 1-hydroxynaphthalene on depression prevalence, with a special focus on the mediating role of inflammatory factors. Using National Health and Nutrition Examination Survey (NHANES) data from 2005 to 2016, this study assessed how 1-hydroxynaphthalene affects depression prevalence through various statistical techniques. The investigation began with restricted cubic spline (RCS) analysis to explore the relationship between 1-hydroxynaphthalene levels and depression prevalence. Logistic regression was then used to examine associations within polycyclic aromatic hydrocarbon (PAH) mixtures , alongside the Bayesian Kernel Machine Regression model. Furthermore, the Quantile G-Computation (QG-comp) model was used to evaluate the influence weights and directions. A mediation analysis was also performed to assess the mediating role of inflammatory factors on the relationship between 1-hydroxynaphthalene and depression prevalence. The study, involving 7813 participants, found a significant J-shaped association between 1-hydroxynaphthalene exposure and depression prevalence (OR (95% CI) = 14.4579 (5.8118–35.9665), *P* < .0001), with 1-hydroxynaphthalene showing the strongest positive contribution to depression prevalence within the phthalate mixture. Inflammatory factors were identified as mediators in the link between 1-hydroxynaphthalene exposure and depression. The findings highlight a significant association between 1-hydroxynaphthalene exposure and depression prevalence, with inflammatory factors acting as mediators. This suggests that inflammatory factors could mediate the harmful effects of environmental pollutants on depression prevalence.

## 1. Introduction

Depression impacts millions globally, affecting health and the economy.^[1]^ Around 4.7% of people worldwide experience depressive episodes annually,^[2]^ reducing productivity and increasing costs.^[2]^ The WHO predicts depression will soon be the leading cause of non-fatal health issues.^[3]^ Severe cases have a 37.3% disability rate and are expected to be the most burdensome disease by 2030.^[4]^

Polycyclic aromatic hydrocarbons (PAHs), containing 2 or more benzene rings, are common environmental pollutants from incomplete combustion of organic materials. Evidence shows PAH exposure increases the risk of cardiovascular disease, diabetes, thyroid, reproductive, and lung function alterations, and cancers like lung cancer.^[5–10]^ Recent studies link PAH exposure to neurobehavioral disorders. A study of 258 children in Taiyuan, China, found maternal PAH exposure affected neurobehavioral development.^[11]^ The New York City cohort study of 253 mother-child pairs suggested PAH exposure during pregnancy increased depression risk in children.^[12]^ Another study found a positive link between urinary PAHs and depressive symptoms in females.^[13]^ The relationship between PAH exposure and depression in adults is still largely unexplored.

Additionally, depression is linked to inflammation.^[14]^ Research shows that chronic stress-induced inflammation affects depression by altering neurotransmitters like serotonin and dopamine through cytokines such as interleukin-1 and tumor necrosis factor-α.^[15]^ Recent studies indicate that blood-derived inflammatory markers, like the platelet-lymphocyte ratio (PLR) and neutrophil-lymphocyte ratio, can be biomarkers for depression.^[16]^ However, the role of inflammation in phthalate-related depression is underexplored.

This study aimed to investigate the effect of 1-hydroxynaphthalene exposure on depression occurrence by analyzing inflammatory mediators using National Health and Nutrition Examination Survey (NHANES) data from 2005 to 2016.

## 2. Method

### 2.1. Study population

The NHANES, managed by the National Center for Health Statistics under the CDC, conducts biennial surveys assessing the health and nutrition of U.S. civilians. This program employs a detailed, multi-stage sampling strategy focusing on a diverse, noninstitutionalized demographic, segmented by age, sex, ethnicity, and socioeconomic status, with data releases every 2 years.

### 2.2. Ethic approval

Informed consent was obtained from all participants. Ethic approval received from National Center for Health Statistics Ethics Review Board (Protocol #2011-17 and Protocol #2005-06).

### 2.3. Inclusion and exclusion criteria

**Figure 1.**
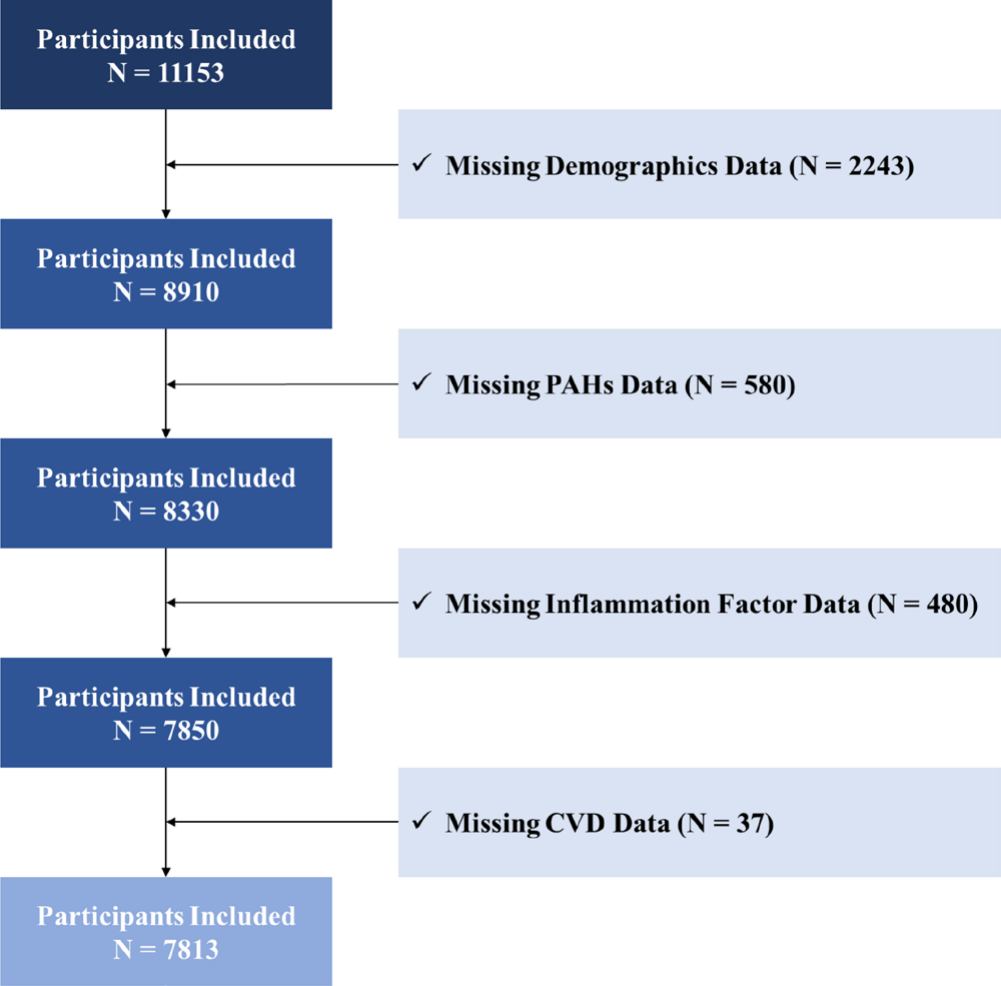
Study flowchart.

Initially, 11,153 participants were enrolled, where 3340 were excluded because of missing data, thus finally including 7813 participants in our study (Fig. 1).

### 2.4. Exposure assessment

This method measures monohydroxylated metabolites of PAHs (OH-PAHs), including 1-hydroxynaphthalene, 2-hydroxynaphthalene, 2-hydroxyfluorene, 3-hydroxyfluorene, 1-hydroxyphenanthrene, 2- & 3-hydroxyphenanthrene, and 1-hydroxypyrene. The procedure involves enzymatic hydrolysis of glucuronidated/sulfated OH-PAH metabolites in urine, extraction via on-line solid phase extraction, and quantification using isotope dilution HPLC-MS/MS.

### 2.5. Outcome assessment

The Patient Health Questionnaire (PHQ-9), a nine-item tool, evaluated depression by assessing symptom frequency over the past 2 weeks and the impact of symptoms. Responses ranged from “not at all” to “nearly every day,” scored 0 to 3.^[17–19]^ A PHQ-9 score of ≤10 indicated no depression, while scores >10 suggested depression.^[17]^

### 2.6. Covariates

Our research included key covariates from previous studies,^[20,21]^ such as demographics (age, gender, race/ethnicity), education, marital status, poverty income ratio, and physical parameters (weight, height, body mass index).

NHANES categorizes race/ethnicity into groups like Mexican American, Other Hispanic, and various Non-Hispanic groups, combining Non-Hispanic Asians with Multi-racial individuals. Educational levels range from below 9th grade to post-college, while marital status ranges from married to unspecified. Poverty income ratio compares household income to adjusted poverty thresholds based on family size.

### 2.7. Statistical analysis

Our statistical analysis used the Kruskal–Wallis method for continuous variables and Fisher exact test for rare categorical data, with PHAs log-transformed for normalization.

We initially applied restricted cubic spline (RCS) to examine the correlation between individual PHAs and depression. Further analysis of PHAs combinations utilized logistic regression, Bayesian Kernel Machine Regression (BKMR) for non-linear interactions, and the QG-comp model to assess impact and directionality.

Mediation analysis with nonparametric bootstrapping (n = 1000) determined direct and indirect effects, and multiple mediation analysis explored potential inflammatory mediators in depression.

To more robustly estimate the potential causal effects of environmental exposure factors on depression levels, this study employed a double machine learning method for cross-sectional data modeling analysis. This method effectively reduces model mis specification bias while controlling for high-dimensional covariates, thereby enhancing the accuracy and stability of causal inference.

We used 1-hydroxynaphthalene as the primary treatment variable (i.e., exposure factor), with depression levels as the outcome variable. Covariates included multiple confounding factors such as demographic characteristics and health behaviors: gender, age, race, education level, marital status, household poverty ratio, alcohol use, hypertension, diabetes, and smoking status.

The analysis process is divided into 2 stages: In the first stage, 6 machine learning algorithms (elastic net, k-nearest neighbors, neural networks, random forests, support vector regression, and XGBoost) are used to fit the treatment variable and outcome variable separately, yielding the residuals predicted by the covariates for the treatment variable and outcome variable; In the second stage, the residuals obtained in the first stage were used to construct orthogonal causal estimates, calculating the marginal effect estimates (Theta), standard errors, 95% confidence intervals, and *P*-values of the exposure variables on the outcome variables.

Each treatment variable was cross-validated with all model combinations, resulting in 36 model combinations (6 first-stage models × 6 second-stage models), ensuring the robustness and consistency of the estimation results. All models are trained and residual estimates are calculated using a 5-fold cross-validation strategy. The results are output and saved in tabular form for subsequent comparison and interpretation.

This method combines the advantages of machine learning in fitting nonlinear and complex high-dimensional data with the requirements of causal inference for robustness and interpretability, offering promising applications in environmental epidemiological research.

Subgroup evaluations confirmed findings across different groups. All statistical methods adjusted for demographic factors^[22,23]^ and were conducted using R software version 4.3.3, with significance set at *P* < .05.

## 3. Result

### 3.1. General information

This study included 7813 subjects, divided into 2 groups based on depression prevalence, with a diagnosis rate of 6.66% (Table 1). Significant differences (*P* < .05) were found between the groups in gender, race/ethnicity, education, marital status, hypertension, diabetes, smoking, all PAHs except 1-hydroxypyrene, and all inflammatory markers except WBC.

**Table 1:** General information.

	Non-depression	Depression	*P*
Population	7293	520	
Gender			
Male	3698 (50.71%)	188 (36.15%)	**.0000**
Female	3595 (49.29%)	332 (63.85%)	
Age	48.64 ± 17.76	48.86 ± 15.91	.7686
Race and ethnicity			
Mexican American	1146 (15.71%)	87 (16.73%)	**.0010**
Other Hispanic	643 (8.82%)	68 (13.08%)	
Non-Hispanic White	3334 (45.72%)	231 (44.42%)	
Non-Hispanic Black	1454 (19.94%)	104 (20%)	
Other Race - including multi-racial	716 (9.82%)	30 (5.77%)	
Educational background			
<9th grade	671 (9.2%)	79 (15.19%)	**.0000**
9–11th grade (includes 12th grade with no diploma)	962 (13.19%)	112 (21.54%)	
High school graduate/GED or equivalent	1679 (23.02%)	135 (25.96%)	
Some college or AA degree	2151 (29.49%)	137 (26.35%)	
College graduate or above	1830 (25.09%)	57 (10.96%)	
Marital status			
Married	3934 (53.94%)	187 (35.96%)	**.0000**
Widowed	529 (7.25%)	46 (8.85%)	
Divorced	739 (10.13%)	92 (17.69%)	
Separated	198 (2.71%)	32 (6.15%)	
Never married	1321 (18.11%)	113 (21.73%)	
Living with partner	572 (7.84%)	50 (9.62%)	
PIR	2.64 ± 1.63	1.67 ± 1.34	**.0000**
Drinking			
No	1992 (27.31%)	138 (26.54%)	.3073
Yes	5301 (72.69%)	382 (73.46%)	
Hypertension			
No	4829 (66.28%)	266 (51.15%)	**.0000**
Yes	2459 (33.72%)	254 (48.85%)	
Diabetes			
No	6466 (88.66%)	401 (77.12%)	**.0000**
Yes	827 (11.34%)	119 (22.88%)	
Smoking			
No	4085 (56.01%)	199 (38.27%)	**.0000**
Yes	3208 (43.99%)	321 (61.73%)	
PAHs			
1-Hydroxynaphthalene	0.3 ± 0.11	0.36 ± 0.14	**.0000**
2-Hydroxynaphthalene	0.45 ± 0.14	0.51 ± 0.15	**.0000**
3-Hydroxyfluorene	0.42 ± 0.17	0.5 ± 0.2	**.0132**
2-Hydroxyfluorene	0.46 ± 0.15	0.53 ± 0.17	**.0008**
1-Hydroxyphenanthrene	0.44 ± 0.12	0.48 ± 0.12	**.0033**
1-Hydroxypyrene	0.43 ± 0.13	0.48 ± 0.14	.3630
Inflammation factors			
WBC	0.37 ± 0.07	0.39 ± 0.07	.9205
Lym	0.68 ± 0.09	0.67 ± 0.09	**.0000**
Mono	0.59 ± 0.07	0.58 ± 0.08	**.0000**
Neu	0.8 ± 0.08	0.81 ± 0.08	**.0000**
Eos	0.55 ± 0.12	0.55 ± 0.12	**.0000**
Baso	0.44 ± 0.15	0.44 ± 0.15	**.0000**

Bold value indicates *P* < .05. PAH = polycyclic aromatic hydrocarbon, PIR = poverty income ratio.

### 3.2. Association between single PAHs and the prevalence of depression

**Figure 2.**
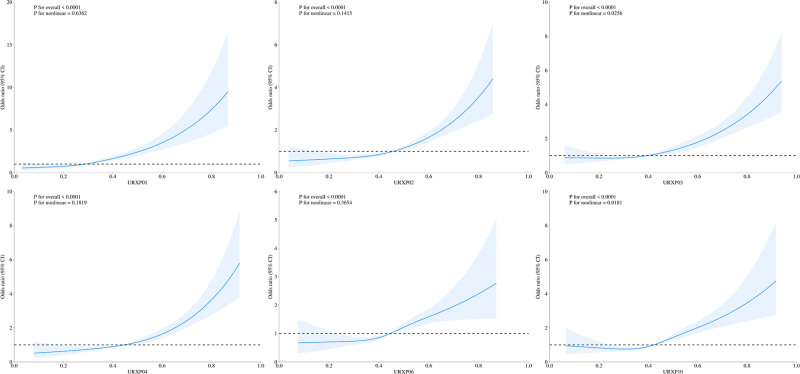
Association between single PAH and depression assessed by RCS model (unadjusted). PAH = polycyclic aromatic hydrocarbon, RCS = restricted cubic spline, URXP01 = 1-hydroxynaphthalene, URXP02 = 2-hydroxynaphthalene, URXP03 = 3-hydroxyfluorene, URXP04 = 2-hydroxyfluorene, URXP06 = 1-hydroxyphenanthrene, URXP10 = 1-hydroxypyrene.

A restricted cubic spline model assessed the association between 1-hydroxynaphthalene and depression prevalence. The initial unadjusted model showed a J-shaped correlation with different relationships across phthalates (*P* < .0001) (Fig. 2). This association remained after adjusting for all covariates (*P* < .0001) (Fig. S1, Supplemental Digital Content, https://links.lww.com/MD/Q504).

### 3.3. Association between 1-hydroxynaphthalene and the prevalence of depression in PAHs mixture assessed by logistic regression model

Logistic regression analyzed the relationship between 1-hydroxynaphthalene and depression incidence within a PAH mixture. 1-Hydroxynaphthalene showed a significant positive correlation with depression rates (OR (95% CI) = 14.4579 (5.8118–35.9665), *P* < .0001) (Table 2). This association remained significant after adjusting for all covariates (OR (95% CI ) = 10.7526 (4.0277–28.7055), *P* < .0001) (Table S1, Supplemental Digital Content, https://links.lww.com/MD/Q505).

**Table 2:** Association between mono-(2-ethyl-5-hydroxyhexyl) phthalate in phthalate mixture and depression assessed by logistic regression model (unadjusted).

Variables	Single factor analysis					Multiple logistic analysis				
	β	S.E	*Z*	*P*	OR (95% CI)	β	S.E	Z	*P*	OR (95% CI)
1-Hydroxynaphthalene	3.8749	0.3431	11.296	**<.0001**	48.1801 (24.5958–94.3789)	2.6712	0.465	5.7448	**<.0001**	14.4579 (5.8118–35.9665)
2-Hydroxynaphthalene	3.0129	0.3247	9.28	**<.0001**	20.3466 (10.7680–38.4458)	0.5372	0.491	1.0941	.2739	1.7111 (0.6537–4.4791)
3-Hydroxyfluorene	2.4865	0.2458	10.115	**<.0001**	12.0193 (7.4238–19.4595)	−3.237	0.9816	−3.298	**.001**	0.0393 (0.0057–0.2690)
2-Hydroxyfluorene	3.1441	0.2903	10.831	**<.0001**	23.1978 (13.1328–40.9766)	5.6056	1.2729	4.4037	**<.0001**	271.9570 (22.4375–3296.3013)
1-Hydroxyphenanthrene	2.5068	0.3766	6.6564	**<.0001**	12.2655 (5.8631–25.6593)	−2.926	0.7225	−4.05	**<.0001**	0.0536 (0.0130–0.2208)
1-Hydroxypyrene	3.0343	0.3354	9.0477	**<.0001**	20.7856 (10.7721–40.1073)	1.7784	0.6535	2.7215	**.0065**	5.9202 (1.6448–21.3086)

Bold value indicates *P* < .05.

CI = confidence interval, OR = odds ratio, SE = standard error.

### 3.4. Association between 1-hydroxynaphthalene and the prevalence of depression in PAHs mixture assessed by BKMR model

**Figure 3.**
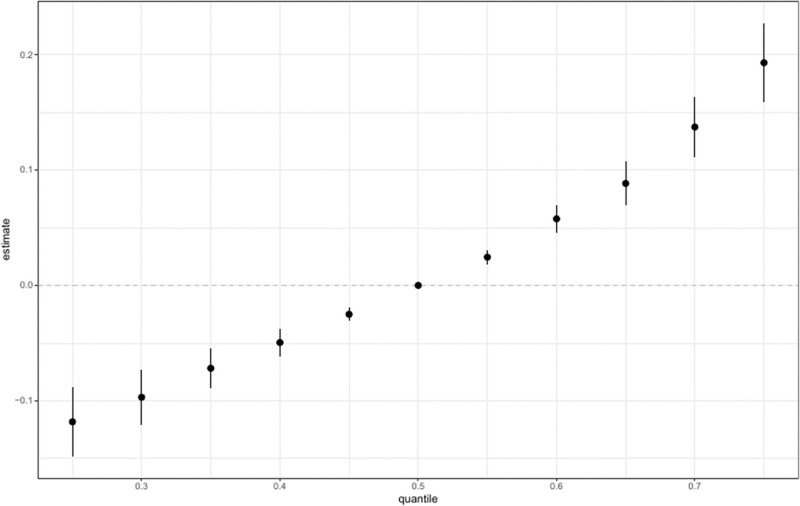
Overall effect of PAH mixtures on the prevalence of depression. PAH = polycyclic aromatic hydrocarbon.

**Figure 4.**
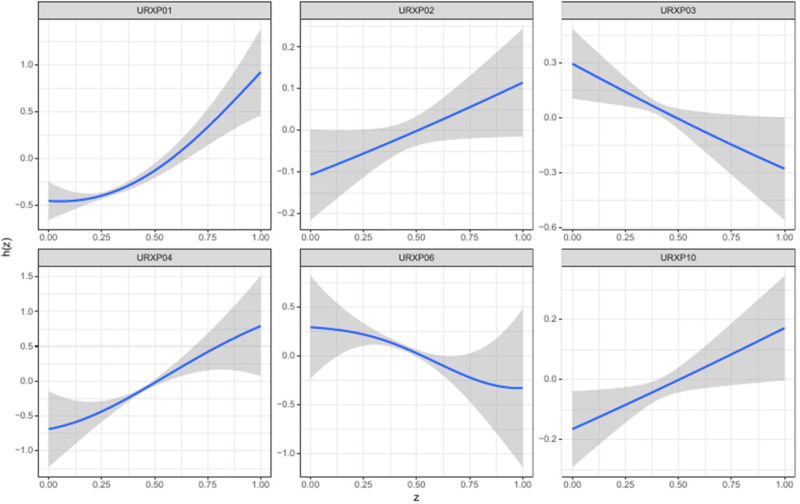
Univariate exposure–response function between each PAH and the prevalence of depression when the other PAH were fixed at 50th percentiles. PAH = polycyclic aromatic hydrocarbon, URXP01 = 1-hydroxynaphthalene, URXP02 = 2-hydroxynaphthalene, URXP03 = 3-hydroxyfluorene, URXP04 = 2-hydroxyfluorene, URXP06 = 1-hydroxyphenanthrene, URXP10 = 1-hydroxypyrene.

**Figure 5.**
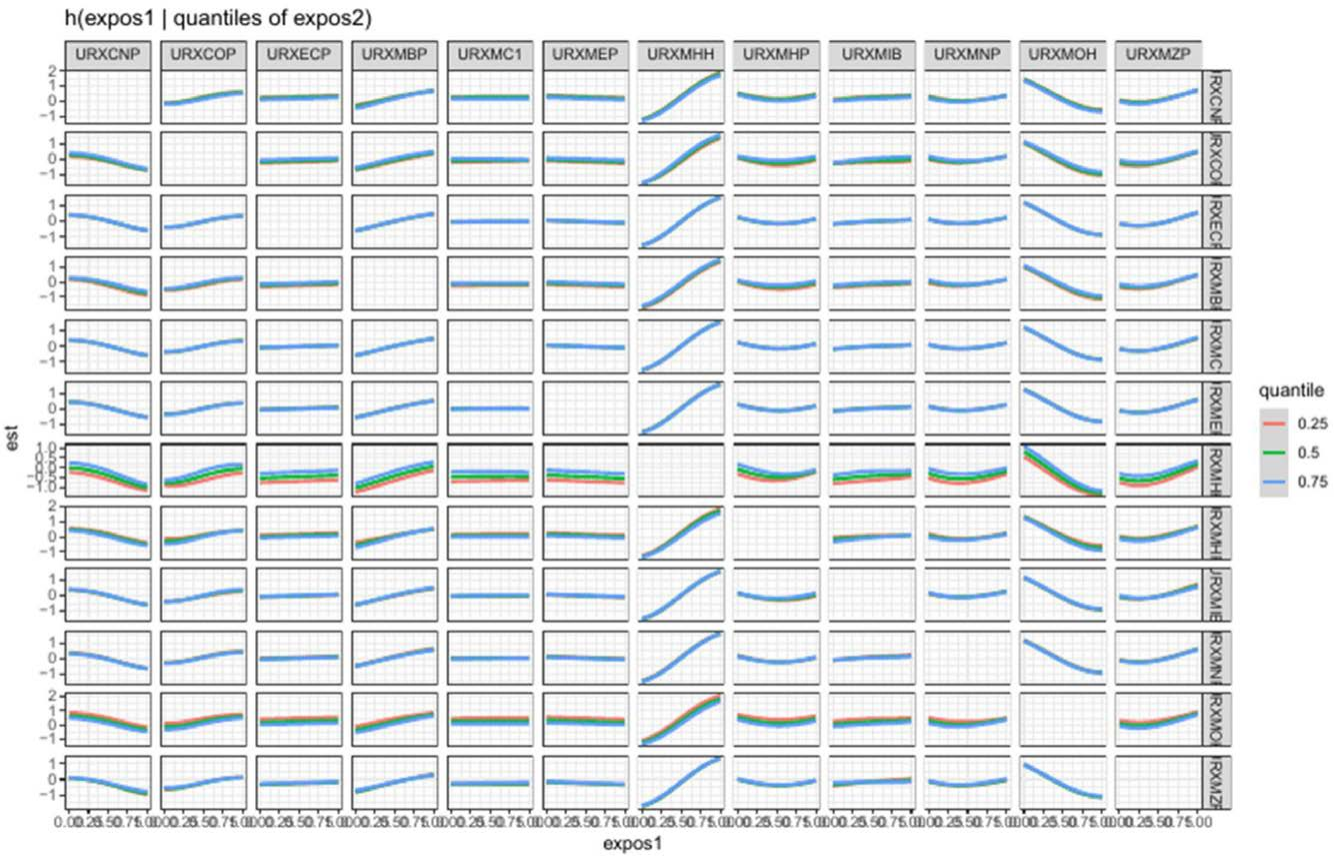
Single exposure – response functions for each PAH and the prevalence of depression when a PAH was at the 75th compared with the 50th percentile and the concentrations of all the other PAH were fixed at either the 25th, 50th, 75th percentile. PAH = polycyclic aromatic hydrocarbon, URXP01 = 1-hydroxynaphthalene, URXP02 = 2-hydroxynaphthalene, URXP03 = 3-hydroxyfluorene, URXP04 = 2-hydroxyfluorene, URXP06 = 1-hydroxyphenanthrene, URXP10 = 1-hydroxypyrene.

**Figure 6.**
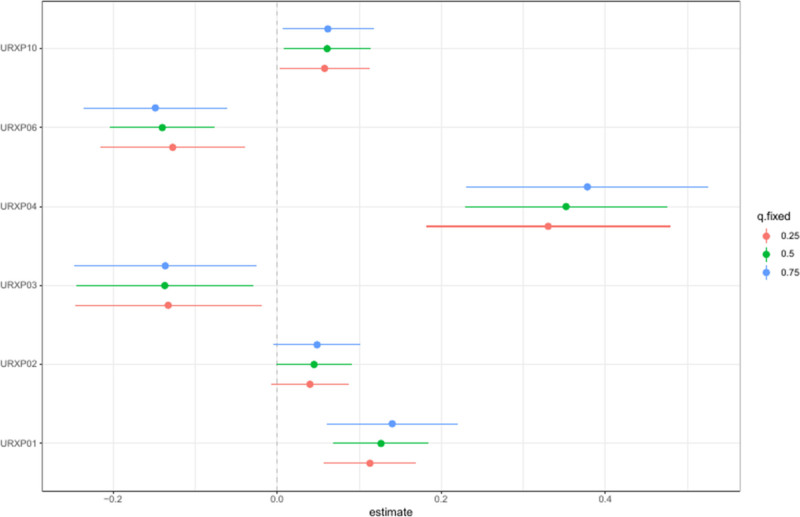
Bivariate exposure – response functions for each PAH and the prevalence of depression when one PAH was fixed at 25th, 50th, 75th percentiles and other PAHs were fixed at the median. PAH = polycyclic aromatic hydrocarbon, URXP01 = 1-hydroxynaphthalene, URXP02 = 2-hydroxynaphthalene, URXP03 = 3-hydroxyfluorene, URXP04 = 2-hydroxyfluorene, URXP06 = 1-hydroxyphenanthrene, URXP10 = 1-hydroxypyrene.

The BKMR method analyzed the relationship between 1-hydroxynaphthalene in a phthalate mixture and depression prevalence. Results showed a positive effect of the PAH mixture on depression rates (Fig. 3). With other PAH levels at their medians, 1-hydroxynaphthalene exhibited a J-shaped correlation with depression incidence (Fig. 4). No significant interaction effects were found (Fig. 5). Setting one phthalate at the 25th, 50th, and 75th percentiles while others stayed at their median also showed a positive association for 1-hydroxynaphthalene with depression (Fig. 6). This PAH had the highest posterior inclusion probabilities among the mix, strongly linking it with depression prevalence (1.00).

### 3.5. Weights and effect direction of 1-hydroxynaphthalene on the prevalence of depression assessed by QG-comp model

**Figure 7.**
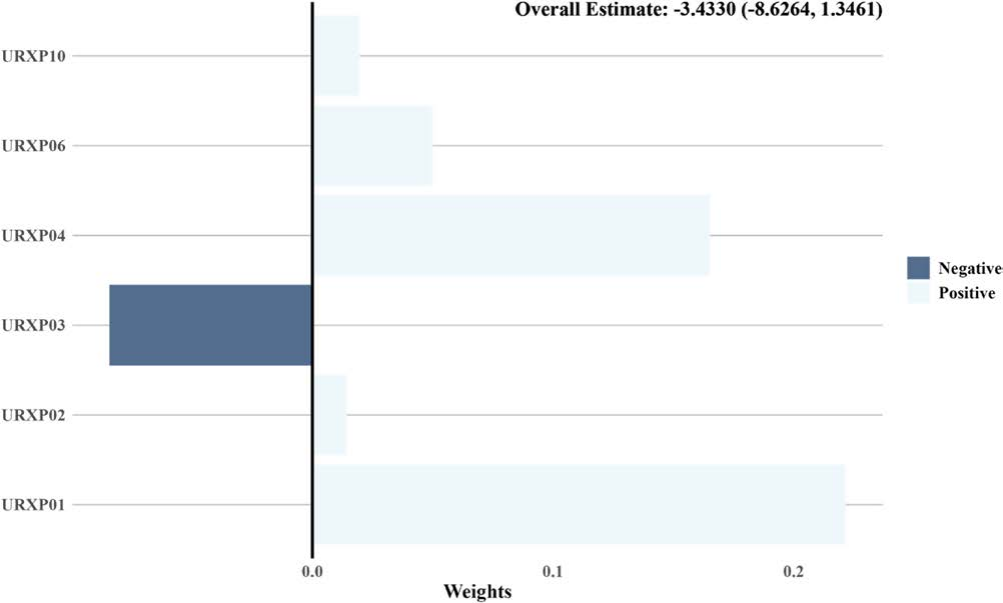
Weights and effect direction of 1-hydroxynaphthalene in PAHs mixture on depression. PAH = polycyclic aromatic hydrocarbon, URXP01 = 1-hydroxynaphthalene, URXP02 = 2-hydroxynaphthalene, URXP03 = 3-hydroxyfluorene, URXP04 = 2-hydroxyfluorene, URXP06 = 1-hydroxyphenanthrene; URXP10 = 1-hydroxypyrene.

The QG-comp model was used to evaluate the influence and directional impact of 1-hydroxynaphthalene on depression prevalence within a PAHs mixture. Figure 7 showed that 1-hydroxynaphthalene had the most substantial positive contribution to the prevalence of depression.

### 3.6. Mediated effect of inflammation factors on the association between 1-hydroxynaphthalene and the prevalence of depression

Mediation analysis was conducted to determine the role of inflammatory markers in mediating the relationship between 1-hydroxynaphthalene and depression prevalence. The WBC was identified as mediators, with mediation percentages of 3.86%.

### 3.7. Association between 1-hydroxynaphthalene and depression in different subgroups

**Figure 8.**
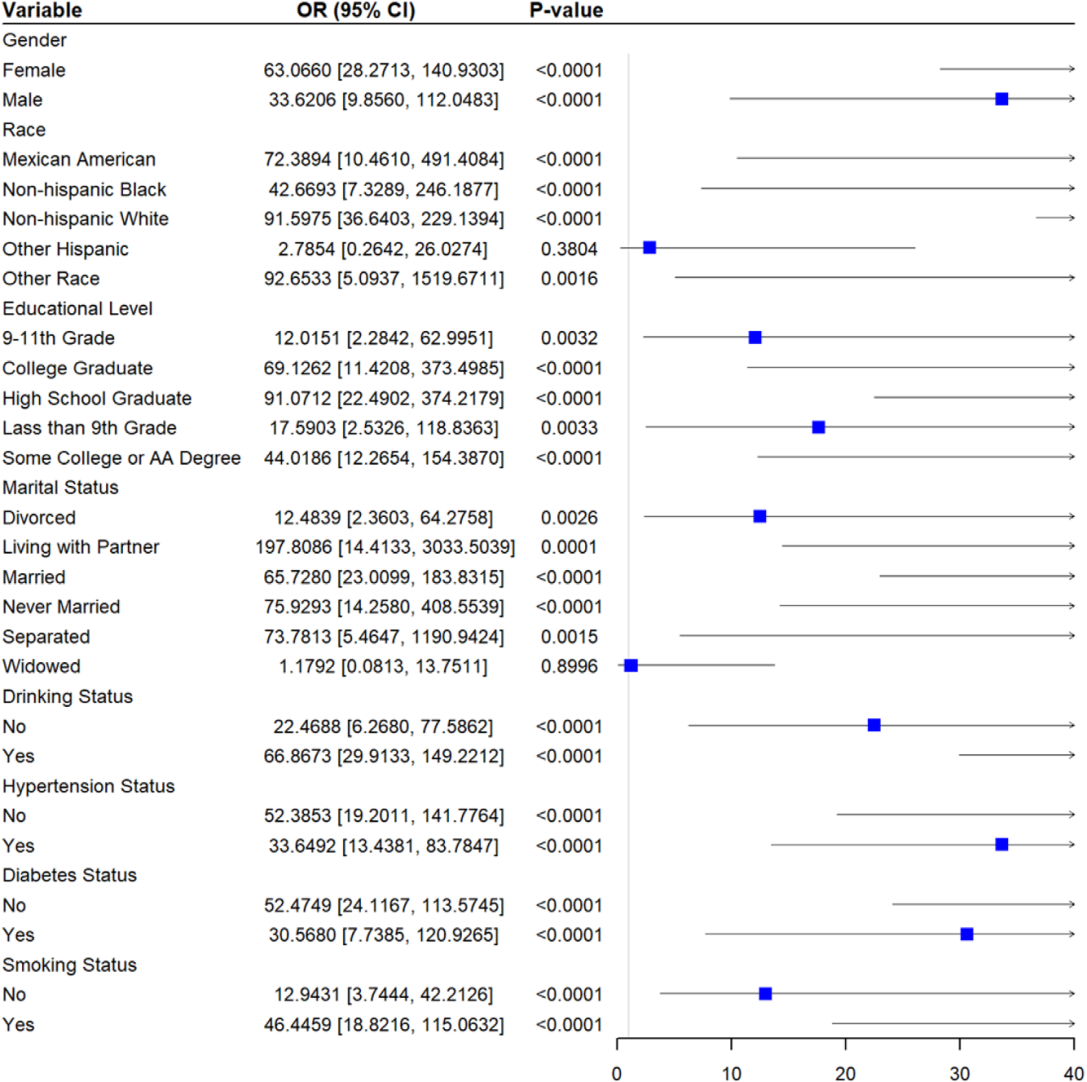
Association between 1-hydroxynaphthalene and the prevalence in different subgroups.

Across various subgroups, the significant positive correlation between 1-hydroxynaphthalene and depression prevalence persisted in almost all subgroups except for population of other race or widowed (Fig. 8).

### 3.8. Causal effect

Among various machine learning models, 1-ydroxynaphthalene has a statistically significant causal association with the onset of depression (Table 3).

**Table 3:** Casual effect.

First stage	Second stage	Theta	StdErr	95% CI lower	95% CI upper	*P*-value
ElasticNet	ElasticNet	0.2744	0.0242	0.2271	0.3217	<.0001
ElasticNet	KNN	0.1843	0.0259	0.1336	0.2350	<.0001
ElasticNet	MLP	0.2031	0.0242	0.1558	0.2505	<.0001
ElasticNet	RFM	0.1642	0.0251	0.1151	0.2133	<.0001
ElasticNet	SVR	0.2808	0.0242	0.2333	0.3283	<.0001
ElasticNet	XGBoost	0.1816	0.0260	0.1305	0.2326	<.0001
KNN	ElasticNet	0.1825	0.0242	0.1351	0.2298	<.0001
KNN	KNN	0.2072	0.0257	0.1568	0.2577	<.0001
KNN	MLP	0.1737	0.0241	0.1265	0.2210	<.0001
KNN	RFM	0.1729	0.0250	0.1240	0.2218	<.0001
KNN	SVR	0.1849	0.0242	0.1374	0.2325	<.0001
KNN	XGBoost	0.2004	0.0259	0.1496	0.2512	<.0001
MLP	ElasticNet	0.1969	0.0248	0.1483	0.2454	<.0001
MLP	KNN	0.1708	0.0264	0.1190	0.2226	<.0001
MLP	MLP	0.2633	0.0246	0.2151	0.3116	<.0001
MLP	RFM	0.1736	0.0256	0.1235	0.2238	<.0001
MLP	SVR	0.1831	0.0248	0.1344	0.2318	<.0001
MLP	XGBoost	0.1810	0.0266	0.1289	0.2331	<.0001
RFM	ElasticNet	0.1601	0.0251	0.1110	0.2093	<.0001
RFM	KNN	0.1717	0.0267	0.1193	0.2241	<.0001
RFM	MLP	0.1695	0.0250	0.1205	0.2185	<.0001
RFM	RFM	0.1939	0.0259	0.1432	0.2446	<.0001
RFM	SVR	0.1677	0.0252	0.1184	0.2171	<.0001
RFM	XGBoost	0.2112	0.0269	0.1585	0.2638	<.0001
SVR	ElasticNet	0.2273	0.0258	0.1766	0.2779	<.0001
SVR	KNN	0.1964	0.0276	0.1424	0.2504	<.0001
SVR	MLP	0.2273	0.0257	0.1768	0.2777	<.0001
SVR	RFM	0.1981	0.0267	0.1458	0.2504	<.0001
SVR	SVR	0.2274	0.0259	0.1766	0.2782	<.0001
SVR	XGBoost	0.2121	0.0277	0.1577	0.2664	<.0001
XGBoost	ElasticNet	0.1624	0.0247	0.1140	0.2108	<.0001
XGBoost	KNN	0.1742	0.0263	0.1227	0.2258	<.0001
XGBoost	MLP	0.1679	0.0246	0.1197	0.2161	<.0001
XGBoost	RFM	0.1856	0.0255	0.1357	0.2355	<.0001
XGBoost	SVR	0.1668	0.0248	0.1183	0.2154	<.0001
XGBoost	XGBoost	0.2197	0.0264	0.1679	0.2715	<.0001

CI = confidence interval.

## 4. Discussion

Few studies have examined the link between PAH exposure and depression risk, despite known health effects of PAHs.^[24]^ Toxicology research shows that prenatal and neonatal PAH exposure can cause neurodevelopmental and behavioral effects like depression.^[25,26]^ The Columbia Center for Children’s Environmental Health study found a positive association between PAH exposure and depression symptoms in children aged 6 to 7 years.^[12]^ Previous studies also linked cigarette smoking, a major PAH source,^[27]^ with a higher depression risk.^[28]^ Our study identified significant positive associations between urinary PAH metabolites and depression risk in US adults, especially smokers. Both logistic and WQS regression indicated that PAH exposure might raise depression risk. Thus, reducing PAH exposure could lower depression risk. Additionally, this study emphasizes the need to consider environmental pollution’s impact on mental health as well as physical health.

Naphthalene, a low molecular weight PAH, is primarily produced during low or moderate temperature combustion processes like smoking.^[27]^ It is metabolized and excreted in urine as 1-OHNa and 2-OHNa. This study found higher urinary PAH metabolite levels in smokers, particularly 1-OHNa and 2-OHNa, with stronger associations between these metabolites and depression in smokers. Our previous research also showed a stronger link between inflammation and these metabolites in smokers than nonsmokers among Chinese urban adults.^[29]^ Zhou et al^[10]^ reported that PAH exposure more significantly impacts lung function in smokers, suggesting that smoking enhances PAH’s adverse health effects. These findings imply that smoking cessation could reduce PAH exposure and its related health risks.

The exact mechanism linking PAH exposure to depression is unclear, but several plausible reasons exist. Studies suggest neuroinflammation may play a role in PAH-related depression.^[30–32]^ Recent animal and epidemiological studies indicate PAHs can increase oxidative stress and cortisol release.^[5,33]^ Hyperactivity of the hypothalamic-pituitary-adrenal axis, which controls stress responses and cortisol levels, is a potential factor in depression.^[34,35]^ PAHs might disrupt this axis, raising depression risk.^[33,34]^ PAH exposure also reduces leukocyte mitochondrial DNA, linked to depression.^[36,37]^ Oxidative stress and mitochondrial DNA damage may mediate PAH-associated depression. PAHs negatively impact the central dopaminergic system and brain structure,^[26,38]^ with dopamine and brain volume changes promoting depression.^[39,40]^ More research is needed to explore these mechanisms.

Inflammation is crucial in depression’s pathogenesis.^[41]^ In depressive patients, systemic immune responses, shown by altered inflammation markers, higher antibody levels, and increased immune cell counts, hinder recovery. A meta-analysis of 82 studies with 3212 MDD patients and 2798 controls found elevated cytokine levels in MDD patients.^[42]^ However, the variability in cytokines and factors like smoking, body mass index, and comorbidities complicate clinical interpretation, leading to a focus on routine tests like blood counts. Meng et al examined 34,324 NHANES participants, revealing complex associations between neutrophil-lymphocyte ratio, PLR, MLR, and depression.^[16]^ A meta-analysis highlighted geographic and study-specific factors affecting these associations, though PLR and MLR are not consistently validated depression markers.^[43,44]^

In view of these mechanisms, it can be supposed that 1-hydroxynaphthalene was associated with the prevalence of depression and this association was mediated by the inflammation factors, which was confirmed by our study.

This study has several limitations. Firstly, NHANES’s cross-sectional design limits establishing causality or long-term effects, requiring further longitudinal studies to explore the phthalates-depression link. Secondly, using the self-reported PHQ-9 scale for diagnosing depression introduces bias. Future research should use multiple diagnostic tools and clinician interviews for accuracy. Thirdly, relying on a single blood sample limits analysis depth; repeated tests could provide more insights. Lastly, while many confounders were adjusted for, additional variables should be considered in future studies to enhance validity.

## 5. Conclusion

Our study found a significant association between 1-hydroxynaphthalene exposure and higher depression rates, with a non-linear impact within PAH mixtures. 1-Hydroxynaphthalene notably increased depression prevalence the most. Inflammatory markers mediated this relationship, underscoring the potential risks.

## Author contributions

**Conceptualization:** Yuzhe Kong.

**Data curation:** Xiaoyi Zhu, Yuzhe Kong.

**Formal analysis:** Qingsong Mao, Xiaoyi Zhu, Yuzhe Kong.

**Investigation:** Xiaoyi Zhu, Yuzhe Kong.

**Methodology:** Yuzhe Kong.

**Project administration:** Yuzhe Kong.

**Resources:** Yuzhe Kong

**Software:** Yuzhe Kong.

**Supervision:** Yuzhe Kong.

**Validation:** Yuzhe Kong.

**Visualization:** Xinyi Zhang, Yuzhe Kong.

**Writing – original draft:** Liuyan Xu, Qingsong Mao, Yuzhe Kong.

**Writing – review & editing:** Liuyan Xu, Yuzhe Kong.

## Supplementary Material




