# Supplementary material for: Mediated effect of white blood cell on the association between 1-hydroxynaphthalene and the prevalence of depression: Evidence from the NHANES (2005–2016)
Source: Medicine (Baltimore). 2025 Nov 14;104(46):e45477. doi: 10.1097/MD.0000000000045477 (PMC12622731; doi:10.1097/MD.0000000000045477)
Supplement: Supplementary file 1 [file medi-104-e45477-s001.pdf]

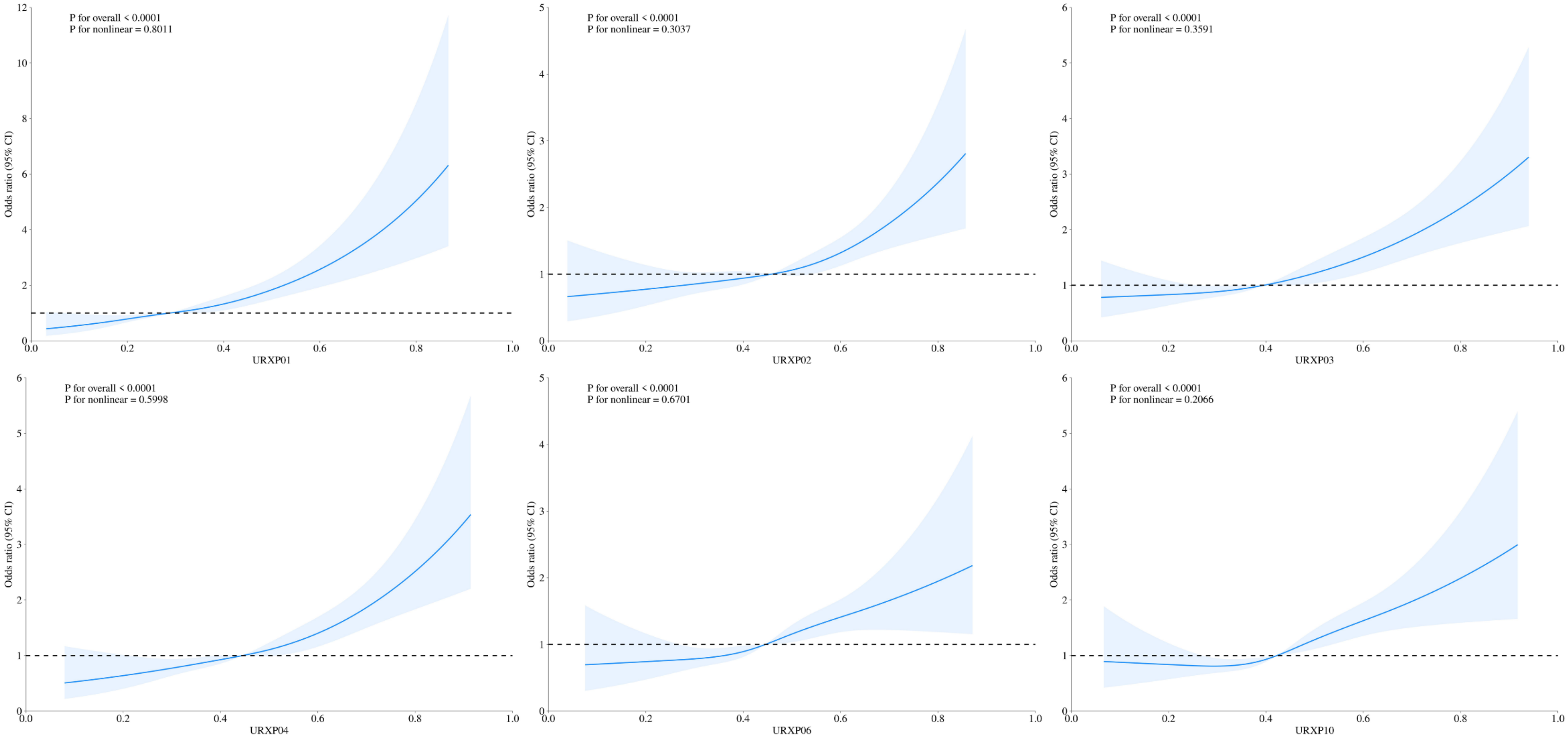

Figure S1 Association between Single Phthalate and Depression Assessed by RCS Model (Adjusted for all Covariates)

Note: URXP01 = 1-Hydroxynaphthalene; URXP02 = 2-Hydroxynaphthalene; URXP03 = 3-Hydroxyfluorene; URXP04 = 2-Hydroxyfluorene; URXP06 = 1-Hydroxyphenanthrene; URXP10 = 1-Hydroxypyrene.
