# Supplementary material for: Mediated effect of white blood cell on the association between 1-hydroxynaphthalene and the prevalence of depression: Evidence from the NHANES (2005–2016)
Source: Medicine (Baltimore). 2025 Nov 14;104(46):e45477. doi: 10.1097/MD.0000000000045477 (PMC12622731; doi:10.1097/MD.0000000000045477)
Supplement: Supplementary file 2 [file medi-104-e45477-s002.pdf]

Table S1 Association between 1-Hydroxynaphthalene in PAHs Mixture and Depression Assessed by Logistic Regression Model

(Adjusted for All Covariates)

Variables	Single Factor Analysis					Multiple Logstic Analysis				
	β	S.E	Z	*P*	OR (95%CI)	β	S.E	Z	*P*	OR (95%CI)
1-Hydroxynaphthalene	3.8749	0.3431	11.296	**<.0001**	48.1801 (24.5958 ~ 94.3789)	2.3751	0.501	4.7408	**<.0001**	10.7526 (4.0277 ~ 28.7055)
2-Hydroxynaphthalene	3.0129	0.3247	9.28	**<.0001**	20.3466 (10.7680 ~ 38.4458)	-0.37	0.5284	-0.7	0.4837	0.6907 (0.2452 ~ 1.9457)
3-Hydroxyfluorene	2.4865	0.2458	10.115	**<.0001**	12.0193 (7.4238 ~ 19.4595)	-2.259	1.0507	-2.15	**0.0316**	0.1045 (0.0133 ~ 0.8192)
2-Hydroxyfluorene	3.1441	0.2903	10.831	**<.0001**	23.1978 (13.1328 ~ 40.9766)	3.9711	1.3371	2.97	**0.003**	53.0430 (3.8592 ~ 729.0467)
1-Hydroxyphenanthrene	2.5068	0.3766	6.6564	**<.0001**	12.2655 (5.8631 ~ 25.6593)	-1.41	0.7718	-1.827	0.0677	0.2442 (0.0538 ~ 1.1082)
1-Hydroxypyrene	3.0343	0.3354	9.0477	**<.0001**	20.7856 (10.7721 ~ 40.1073)	1.1129	0.6954	1.6002	0.1095	3.0430 (0.7787 ~ 11.8921)
